# Delayed Diagnosis of Neck Pain: Eagle Syndrome

**DOI:** 10.7759/cureus.18286

**Published:** 2021-09-25

**Authors:** Shaili Priyamvada, Hukam Singh, Pankul Parnami, Arun Puri, Sukhmanii Kahlon, Akshit Chitkara, Piyush Puri, Yogita Suri

**Affiliations:** 1 Otorhinolaryngology, Safdarjung Hospital, New Delhi, IND; 2 Otolaryngology - Head and Neck Surgery, Rama Medical College Hospital & Research Center, Hapur, IND; 3 Internal Medicine, Jawaharlal Nehru Medical College, Belagavi, IND; 4 Pathology, Adesh Institute of Medical Sciences & Research, Amritsar, IND; 5 Pathology, Government Medical College, Amritsar, Amritsar, IND; 6 Internal Medicine, Medical University of the Americas, Camps Charlestown, KNA; 7 Internal Medicine, University of California Riverside School of Medicine, Los Angeles, USA; 8 Internal Medicine, Rama Medical College Hospital & Research Center, Hapur, IND; 9 Surgery, Saraswathi Institute of Medical Sciences, Hapur, IND

**Keywords:** acute pain, pain, women neck pain, unilateral neck pain, neck pain, eagle syndrome

## Abstract

Eagle syndrome is a rare condition characterized by an abnormally elongated styloid process with or without abnormal direction and/or ossification of the styloid ligament. Clinically, it consists of throat and neck pain radiating to the ear. Here, we present the case of a 34-year-old female with the complaint of left-sided neck pain below the ear for the past year. The patient had tried different analgesics after seeing different doctors, but the pain did not resolve. After conducting radiological investigations at the hospital, a diagnosis of Eagle syndrome was made. The patient was treated with surgical styloidectomy, followed by subsequent remission of the symptoms.

## Introduction

Eagle syndrome was initially described by Watt Weems Eagle who was an American otorhinolaryngologist. It is an abnormality of ossification/morphology of the styloid process [1]. Normally, the length of the styloid process ranges from 25 to 30 mm. If the styloid process is longer than 30 mm, it is considered to be an elongated styloid process. The styloid process is derived from the temporal bone. Between the internal and external carotid arteries lies the apex of the styloid process which is clinically important [2]. The incidence of elongated styloid process ranges from 4-7%, of which only 4% of the patients are symptomatic [3]. Symptoms due to the elongated styloid process are believed to be caused by direct pressure of the styloid process on the nearby nerves and vessels, that is, the facial nerve and the internal and external carotid arteries. In addition to neck pain, otalgia, tinnitus, and dysphagia can also occur. The first step toward the treatment of Eagle syndrome is conservative management using analgesics. If conservative measures fail, the final treatment involves resection of the elongated part of the styloid process.

There are two subtypes of Eagle syndrome, the classical syndrome and the stylocarotid syndrome. Patients with classic Eagle syndrome often present with pain, dysphagia, and foreign body sensation immediately following tonsillectomy, whereas pain, visual disturbances, and syncope due to carotid artery compression are features of the stylocarotid syndrome. However, studies have shown that it can occur without tonsillectomy [4,5].

## Case presentation

A 34-year-old female presented to the hospital with a complaint of left neck pain below the ear for the past year. The pain did not respond to the various analgesics prescribed by other doctors and was a dull aching type of pain that intensified on swallowing.

**Figure 1:**
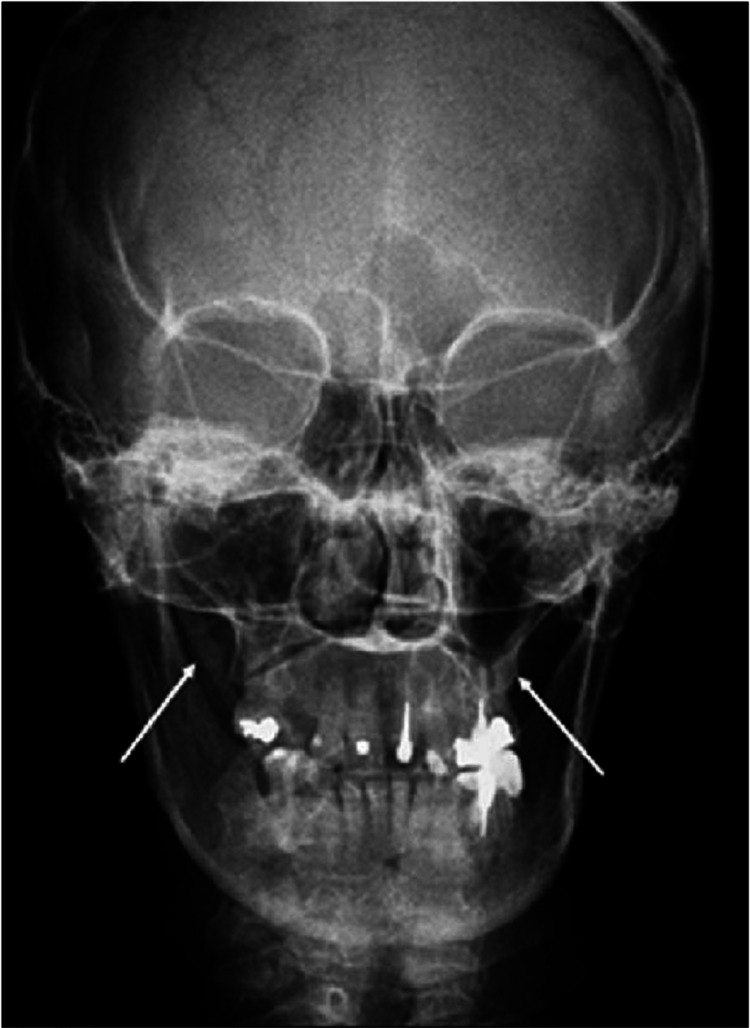
X-ray: reverse Towne’s view showing the elongated styloid process of the patient denoted by white arrows.

Examination of the bilateral ears was normal. On inspection and examination, the oropharynx was normal; however, bimanual palpation of the tonsillar fossae on the left side elicited tenderness. The nose and nasal cavity were normal on examination. Neck examination was also normal. Pure tone audiometry and impedance audiometry were done which were normal as well. In addition, routine blood investigations including complete blood count, liver function tests, and kidney function tests were within normal limits. Figure 1 shows the X-ray (reverse Towne’s view).

**Figure 2:**
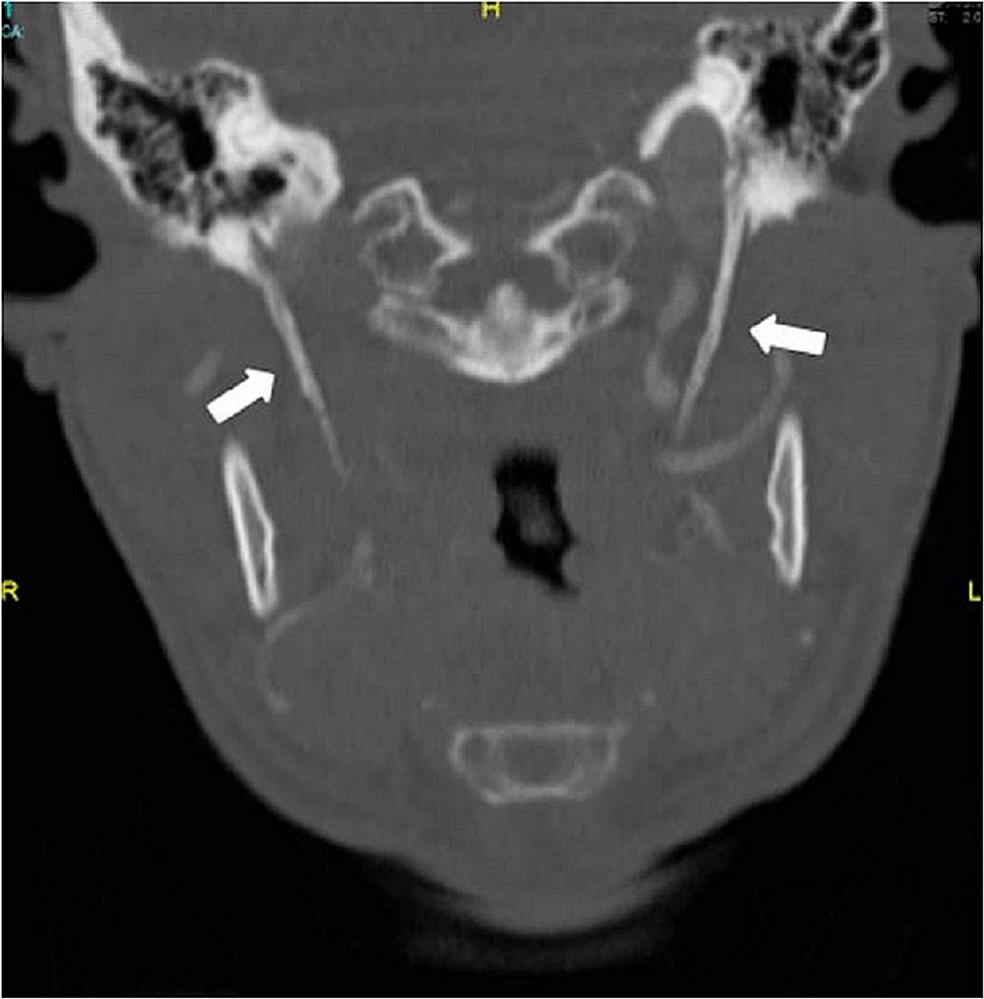
CT scan of the neck: coronal view. Arrows show the elongated styloid process. CT: computed tomography

Computed tomography (CT) scan revealed bilateral elongated styloid processes: right styloid process measured 45 mm and left styloid process measured 48 mm (Figure 2).

**Figure 3:**
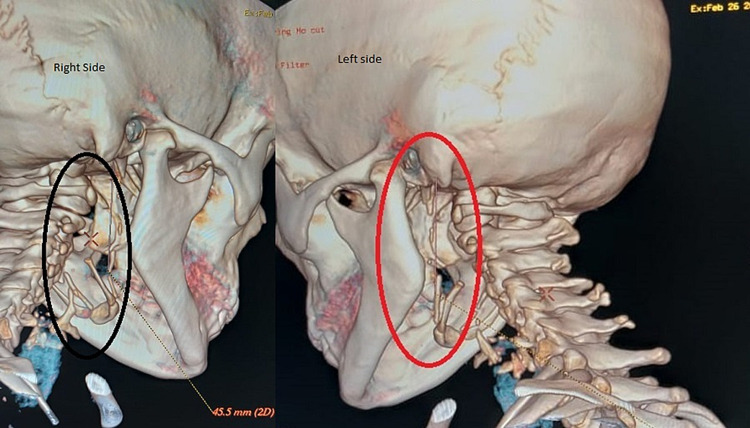
Three-dimensional CT reconstruction showing the elongated right and left elongated styloid process. CT: computed tomography

CT three-dimensional reconstruction showed the elongated styloid process of the right (45 mm) and the left sides (48 mm) (Figure 3).

**Figure 4:**
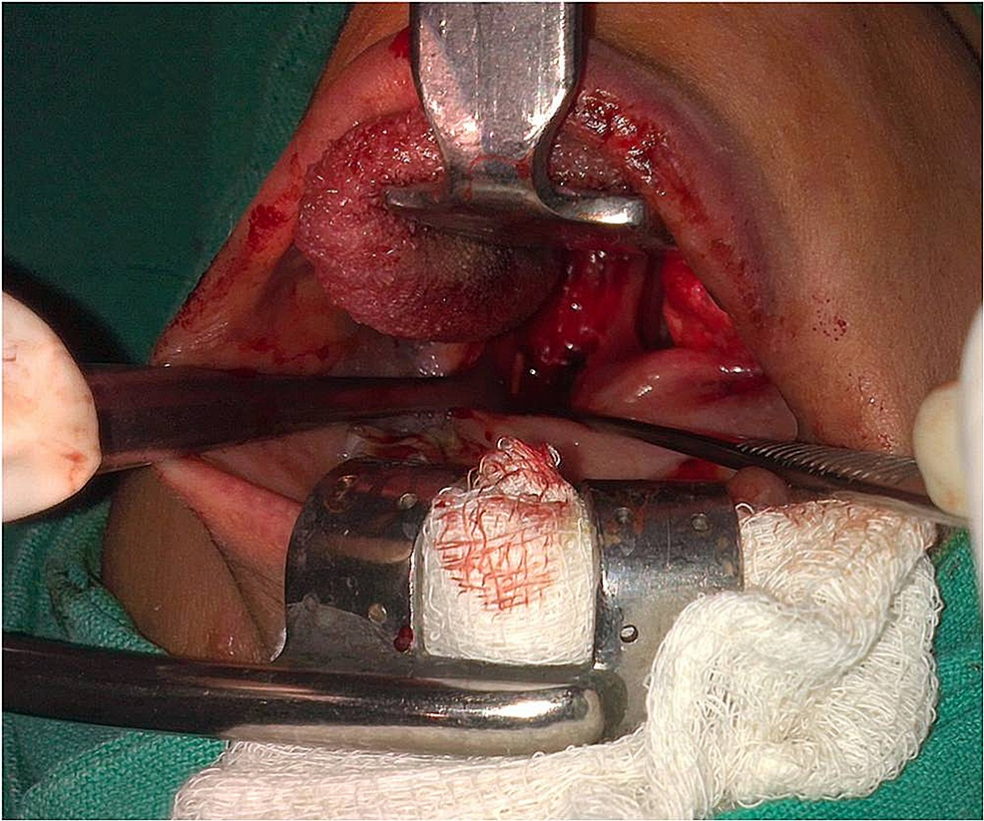
Transoral styloidectomy.

After the diagnosis was confirmed, transoral styloidectomy was performed (Figure 4).

**Figure 5:**
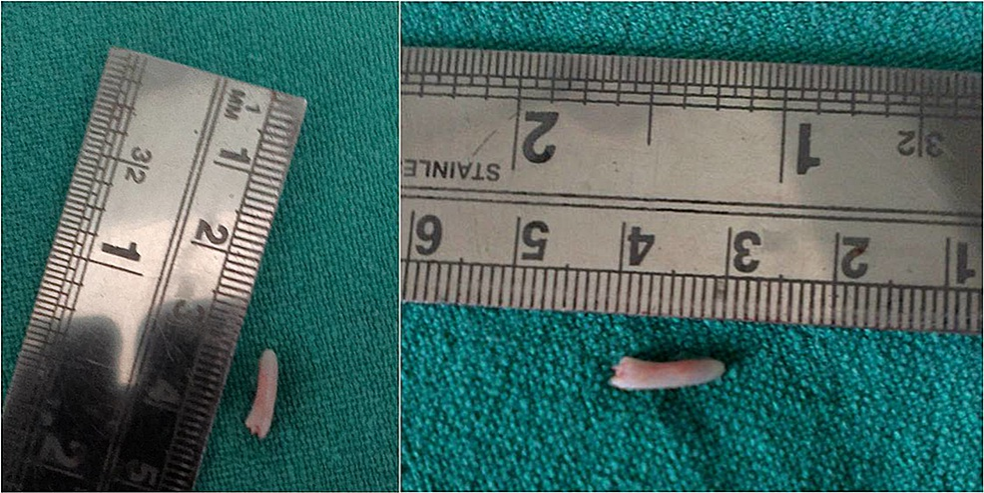
Post-styloidectomy the elongated portion of the styloid processes was removed.

Left transtonsillar styloidectomy was performed, and the symptoms resolved subsequently (Figure 5). After surgery, the pain resolved, and she did not require any anti-inflammatory drugs and regained normal quality of life.

## Discussion

Eagle syndrome or stylocarotid artery syndrome is characterized by an abnormally increased length of the styloid process. It is a form of nerve pain that causes people to experience a dull, throbbing ache around the face and throat. The pathogenesis remains obscure and the symptoms can be variable [6,7]. Symptoms can include a constant dull pharyngeal pain or a sharp shooting pain, but most commonly the latter. Other clinical features include dysphagia, headache, tinnitus, and foreign body sensation in the pharynx [8]. CT usually confirms the diagnosis of the elongated styloid process, although the diagnosis can be made using a plain radiograph [9]. The differential diagnosis for Eagle syndrome includes temporal arteritis, glossopharyngeal and trigeminal neuralgia, cluster headache, migraine, pain related to unerupted third molars, cervical arthritis, tumors, and ill-fitting or missing dentures.

Intraoral transtonsillar and transcervical are two surgical approaches for the treatment of Eagle syndrome. The advantages of the intraoral approach are that it produces no external scarring and has low postoperative morbidity and complications [4,9]. The transcervical approach is performed by an extraoral approach through an incision that extends from the mastoid process along the sternocleidomastoid to the level of the hyoid, and then up across the neck to the midline of the chin. The disadvantage of the transcervical approach is that it is aesthetically unpleasing and has more morbidity compared to the intraoral approach [10-12].

## Conclusions

Our patient visited multiple doctors of various specialties because of the vague dull aching nature of the pain in the neck. Since it recurred, it remained undiagnosed for a long period of time. Patients with Eagle syndrome usually remain undiagnosed which adds to the suffering and cost of treatment for the patient. Eagle syndrome is an easy-to-miss diagnosis, especially in females where dull aching pain is not usually paid significant attention in developing countries like India where it can be considered a functional cause instead of an organic one. Eagle syndrome should always be suspected in the case of idiopathic unilateral craniocervical pain, especially in adult women and when the pain is not responsive to analgesics. Otolaryngologists, neurologists, and dental surgeons should be aware of the existence and incidence of this clinical entity, which is associated with reduced quality of life.
